# Evaluation of the Relationship Between Environmental Factors, Nutrition, and Metabolic Changes in Children Diagnosed With Autism in North Cyprus: A Case-Control Study

**DOI:** 10.7759/cureus.17016

**Published:** 2021-08-09

**Authors:** Lubna Qutranji, Tasnim Alkayyali, Wasef Alkhateeb, Aybuke Sapmaz, Ahmad Aleter, Ahmad Almoustafa, Zeynep Aci, Amer Hammad, Amr Nakhala, Umut Altunc

**Affiliations:** 1 Pediatrics, Eastern Mediterranean University, Faculty of Medicine, Famagusta, CYP; 2 Internal Medicine, Mansoura University, Mansoura, EGY; 3 Pediatrics, Famagusta State Hospital, Famagusta, CYP

**Keywords:** autism, autism spectrum disorder, risk factors, protective factors, epidemiology, north cyprus

## Abstract

Introduction

Autism spectrum disorder (ASD) is a set of neurodevelopmental disorders characterized by deficits in social behaviors and nonverbal interactions. The disorder is believed to be multifactorial regarding etiopathology. This study aimed to investigate the possible risk factors associated with the development of autism in the prenatal and postnatal periods.

Methods

We conducted an unmatched case-control study composed of 56 autistic cases and 85 control children in North Cyprus. Cases recruited were previously diagnosed by a pediatric psychiatrist as being on the autistic spectrum. Parental questionnaires were distributed, and the collected data were analysed using Statistical Package for the Social Sciences (SPSS) version 24 (IBM Corp, Armonk, NY). Binary logistic regression was used to compute the adjusted odds ratios (aOR), adjusted for possible confounders.

Results

Our results showed increased odds of developing ASD in mothers with mental disorders such as depression and anxiety (aOR 6.99; 95% CI 1.94 - 25.24), mothers with medical conditions such as Hashimoto’s thyroiditis (aOR 2.68; 95% CI 1.06 - 6.78), mothers using aluminum-containing anti-acids (aOR 2.34; 95% CI 1.012 - 5.39), mothers exposed to loud noises during pregnancy (aOR 2.66; 95% CI 1.005 - 7.034), mothers with ≥ two previous miscarriages (aOR 4.19; 95% CI 1.17 -14.97), neonates with birth weight <2500 grams (aOR 4.19; 95% CI 1.16 - 14.84), male gender neonates (aOR 3.26; 95% CI 1.31 - 8.90), and neonates exposed to MRI or CT scan during the first year of life (aOR 6.94; 95% CI 1.15 - 42.07). Decreased odds of ASD development were observed in mothers using multivitamins during pregnancy (aOR 0.35; 95% CI 0.13 - 0.97), mothers consuming slight amounts of baking powder during pregnancy (aOR 0.235; 95% CI 0.09 - 0.60), mothers with threatened abortion (aOR 0.35; 95% CI 0.12 - 0.98), and neonates taking iron supplementation during the first six months of life (aOR 0.38; 95% 0.16 - 0.91).

Conclusion

There were various maternal and neonatal factors associated with ASD development in North Cyprus. Although there is some evidence to suggest that exposure to specific factors during prenatal or postnatal periods may increase the risk of ASD, there is insufficient evidence that implicates a specific factor for autism etiology. Future studies are recommended to be performed on larger scales to support further the factors associated with ASD development.

## Introduction

The world health organization estimates that the prevalence of autism spectrum disorder (ASD) is one per 160 children worldwide [1], and the cost of autism reached 268 billion dollars in the United States of America in 2015 [2]. ASD encompasses various conditions manifested by social and language deficits, difficulties in nonverbal communication skills, and stereotyped repetitive behaviors [3]. Signs of ASD usually appear by age two or three; however, developmental delays may occur earlier, allowing the diagnosis to be made as early as 18 months of age [3]. Multiple factors have been implicated in the development of ASD, including environmental, biological, and genetic factors [4].

Despite being a common condition with a lifelong course, ASD showed no apparent connection with a single specific risk factor [2]. Instead, a combination of both genetic and environmental factors is suggested [5]. The previously studied environmental factors in research include maternal age, paternal age, cesarean section, medication use during pregnancy, and prenatal infections [5]. Identifying the possible risk factors linked to ASD is essential in preventing the condition. In addition to the substantial economic burden of ASD, the quality of life for affected individuals and their families is decreased substantially [2]. Therefore, targeting disease prevention is a crucial element in improving the quality of life in recent years.

Many researchers in the literature tried to discover the relationship between ASD and possible risk factors, together with potential protective factors. One meta-analysis study linked ASD development with abnormal fetal presentations, umbilical cord complications, fetal distresses, birth injury or trauma, multiple births, maternal hemorrhage, summer birth, low birth weight, small for gestational age, and congenital malformations [6]. Another meta-analysis included factors such as mother's and father's races, gestational hypertension, gestational diabetes, maternal and paternal education, threatened abortion [7]. A third study focused on the protective factors against ASD development, suggesting that folate, vitamin D, environmental toxin avoidance, correcting vitamin deficiencies, immune boosting, and prolonged breastfeeding might help prevent ASD [8].

Our study investigated the relationship between autism and prenatal, perinatal, and postnatal conditions to suggest possible risk factors and protective factors for ASD. We conducted an unmatched case-control study among children with and without autism in North Cyprus. Because there is no autism registry in North Cyprus, we obtained cases from schools of children with developmental disabilities.

## Materials and methods

Study sample

We conducted a case-control study in the Turkish Republic of Northern Cyprus in 2017 among children under the age of 18. According to the Statistical Yearbook of Northern Cyprus published in February 2016, the total number of children in North Cyprus under 18 years was 73,500, with an average of 4000 new births per year [9]. Approximately 300 children were enrolled in special education centers in North Cyprus, and around 100 of them were diagnosed as having an autism spectrum disorder. Based on the given numbers, we estimated the required sample size of cases as 56 cases using the "openepi" website for unmatched case-control studies [10]. The term "cases" refers to children who were diagnosed as being autistic by a pediatric psychiatrist in North Cyprus, based on the Diagnostic and Statistical Manual of Mental Disorders, 5th Edition (DSM-5) criteria [11]. Most autistic children were not getting any medical treatment, while only 5-10% were under psychotic or epileptic treatment. The term “controls” refers to children without a diagnosis of autism who were enrolled in public schools.

Procedure

We obtained ethical approval from the Ethics Committee of Eastern Mediterranean University. In addition, informed consent was obtained from the parents of autistic children by visiting eight special education centers in North Cyprus and calling the parents of autistic children registered in those centers. We then distributed hard-copy questionnaires to be filled by the mothers of autistic children and collected the questionnaires one month later. We also obtained informed consent from the mothers of controlled children by reaching out to two public schools in North Cyprus and distributing the questionnaires to the heads of those public schools. The questionnaire was distributed in the Turkish language and was developed based on a thorough literature review of similar studies. The questionnaire asked yes-no questions about the possible exposure to each variable during the pregnancy period.

Variables

The demographic variables in our study include gender, child’s age, mother’s and father’s ages at the birth of their child, child’s order, birth weight, birth month, mother’s country of birth, living in an old house, mother having medical conditions, and previous miscarriages. The other part of the survey focused on the possible risk factors or protective factors associated with the development of autism during the prenatal, perinatal, and postnatal periods. The odds ratio was adjusted for potential confounders, including mother’s age, father’s age, child’s gender, birth weight, previous miscarriages, mother’s medical conditions, and pregnancy-induced medical conditions. These confounders were chosen based on data from previous literature studies.

Statistical analysis

Data analysis was done through the Statistical Package for the Social Sciences (SPSS) version 24 software (IBM Corp., Armonk, NY) and statistical significance was set at a two-sided P-value of <0.05. Descriptive statistics were computed on all variables as the primary method of data evaluation. We compared the distribution of demographics and general characteristics between autistic and non-autistic children by performing Pearson’s chi-square test, as well as an independent sample t-test for quantitative variables including child’s age, mother’s age, father’s age, and order of the child. We also used the binary logistic regression analysis with a 95% Confidence Interval (CI) to calculate the unadjusted odds ratios (OR) and the adjusted odds ratios (aOR) for potential confounders. The statistical analysis on SPSS was done using the number of responses received for each variable, and the missing responses were not included in our statistical calculations.

## Results

Demographical characteristics

The total sample size included in our study comprised 141 subjects, 56 cases and 85 controls. The potentially eligible number of autistic cases in North Cyprus was estimated to be 100; out of these cases, we managed to collect responses from the mothers of 56 autistic children, which was the number of cases suggested by “openepi website”. We also distributed 100 questionnaires for the control group, and we managed to collect responses from the mothers of 85 controlled children. Reasons for non-participation in this study included parental preferences, as some parents did not want to feel guilty for potentially causing the disease to their children. The means of ages in cases and controls were 9.6±5.3 and 6.3±2.8, respectively (P<0.0001). In the cases recruited, 78.6% were males, and 21.4% were females, while in the controls recruited, 56.5% were males, and 43.5% were females (P=0.007). These findings reveal the unmatching of ages and genders between the cases and controls in our study. The mean of mothers’ ages at birth was also significantly different between cases (27.8±4.9) and controls (30.9±4.4) with P<0.0001. However, the mean of fathers’ ages showed no significant difference between both groups (P=0.213). The mean order of a child was slightly higher in the cases (1.5±0.7) compared to controls (1.3±0.5) with P<0.049. The birth weight and birth month showed no differences between both groups (P >0.05). Table 1 shows the demographics and general characteristics of participants.

**Table 1:** Demographical Characteristics **: P<0.05 is considered statistically significant. SD: Standard Deviation, CI: Confidence Interval, HTN: Hypertension, GDM: Gestational Diabetes Mellitus, Independent sample t-test calculated for: child’s age, mother’s age, father’s age, and order of the child, Pearson’s chi-square test (X^2^) calculated for the other variables.

Variable	Cases	Control	P-Value	X^2^	t-test	Mean Difference (95% CI of the Mean Difference)	Number of Participants with Missing Data
Number	56	85	-	-	-	-	-
Gender (%)							
Male	44 (78.6%)	48 (56.5%)	0.007**	7.272	-	-	none
Female	12 (21.4%)	37 (43.5%)				-	none
Mean child’s age (SD), in years	9.6 (5.3)	6.3 (2.8)	<0.0001**	-	4.808	3.3 (2.02 to 4.8)	2
Mean mother’s age (SD), in years	27.8 (4.9)	30.9 (4.4)	<0.0001**	-	-3.949	-3.1 (-4.7 to -1.6)	none
Mean father’s age (SD), in years	31.8 (5.7)	32.9 (4.5)	0.213	-	-1.251	-1.1 (-2.8 to 0.6)	2
Mean order of child (SD)	1.5 (0.7)	1.3 (0.5)	0.049**	-	1.988	0.2 (0.001 to 0.4)	1
Birth weight (%)							
<2500g	8 (14.5%)	6 (7%)	0.149	2.08	-	-	1
>2500g	47 (85.5%)	79 (93%)					
Born in winter months (December, January, February) (%)	16 (28.5%)	22 (25.8%)	0.725	0.124	-	-	none
Mother's country of birth different than North Cyprus (%)	11 (20%)	15 (17.6%)	0.765	0.089	-	-	none
Living in a house that was painted >20 years ago (%)	4 (7%)	2 (2.3%)	0.162	1.951	-	-	4
Having a medical condition during pregnancy (e.g. Hashimoto thyroiditis, anemia) (%)	18 (32%)	17 (20%)	0.087	2.934	-	-	6
Having a pregnancy related disease (e.g.: HTN, GDM) (%)	9 (16%)	11 (13%)	0.549	0.358	-	-	5
Risk of miscarriage/bleeding during pregnancy (%)	10 (17.8%)	26 (30.5%)	0.132	2.267	-	-	1
Difficulties at birth (e.g. oxygen deprivation) (%)	9 (16%)	6 (7%)	0.079	3.077	-	-	3

Continuous variables that were categorized in our study included birth weight, which was categorized into <2500g and >2500g; mother’s and father’s ages at delivery, which were categorized into <30 years and >30 years of age; and birth month, which was categorized into winter months (December, January, February) and non-winter months. Potential confounders were chosen based on data from previous literature studies. Adjusted confounders in our study included mother’s age, father’s age, child’s gender, birth weight, previous miscarriages, mother’s medical conditions, and pregnancy-induced medical conditions.

Maternal factors during pregnancy

Several maternal factors have shown statistically significant results concerning the development of autism. Among these maternal factors, increased risk of autism was seen in mothers with mental disorders during pregnancy, such as anxiety and depression (aOR 6.99; 95% CI 1.94 - 25.24), mothers with medical conditions during pregnancy, such as Hashimoto’s thyroiditis and anemia (aOR 2.68; 95% CI 1.06 - 6.78), mothers using aluminum-containing antacids during pregnancy (aOR 2.34; 95% CI 1.012 - 5.39), and mothers exposed to prolonged periods of noise pollution, i.e., loud noises during pregnancy (aOR 2.66; 95% CI 1.005 - 7.034). Decreased risk of autism was seen in mothers taking multivitamins during pregnancy (aOR 0.35; 95% CI 0.13 - 0.97), mothers using baking powder twice a month while cooking (aOR 0.235; 95% CI 0.09 - 0.60), and mothers with threatened abortion (aOR 0.35; 95% CI 0.12 - 0.98). The following maternal factors showed no statistical significance in autism development: maternal smoking during pregnancy, maternal second-hand smoke exposure, pregnancy-induced conditions, such as gestational diabetes and hypertension, maternal vitamin D deficiency, hyperemesis, insecticide exposure, hair dying, consuming seafood and caffeine, heavy metal exposure, such as mercury, maternal vaccination, microwave usage, owning a pet, and medication use during pregnancy (P ≥ 0.05). Table 2 displays the maternal factors.

**Table 2:** Maternal Factors During Pregnancy **: P<0.05 is considered statistically significant. CI: Confidence Interval, HTN: Hypertension, GDM, Gestational Diabetes Mellitus

Risk Factor	Unadjusted Odds Ratio (95% CI)	P-Value	Adjusted Odds Ratio (95%CI)	P-Value
Maternal smoking	3.478 (0.993 to 12.187)	0.051	2.698 (0.665 to 10.948)	0.165
Second-hand smoke exposure	1.775 (0.769 to 4.095)	0.178	1.955 (0.7 to 5.461)	0.201
Mothers with a mental disorder during pregnancy (e.g. anxiety and depression)	6.06 (2.204 to 16.662)	0.0005**	6.993 (1.937 to 25.244)	0.003**
Mothers with a medical condition during pregnancy (e.g. Hashimoto’s thyroiditis, anemia)	1.966 (0.902 to 4.289)	0.089	2.679 (1.059 to 6.776)	0.037**
Mothers with pregnancy related disease (e.g. HTN, GDM)	1.339(0.514 to 3.488)	0.550	1.289 (0.391 to 4.249)	0.676
Vitamin D deficiency in mother	1.339 (0.465 to 3.851)	0.589	1.223 (0.359 to 4.169)	0.748
Threatened abortion	0.531 (0.232 to 1.219)	0.135	0.346 (0.122 to 0.982)	0.046**
Hyperemesis	0.837 (0.394 to 1.775)	0.642	0.525 (0.21 to 1.315)	0.169
Insecticide exposure	1.086 (0.391 to 3.022)	0.874	0.883 (0.268 to 2.912)	0.838
Use of hair dye	0.833 (0.352 to 1.973)	0.678	1.229 (0.397 to 3.801)	0.720
Consumption of seafood	0.681 (0.319 to 1.4535)	0.320	0.53 (0.214 to 1.315)	0.171
Heavy metal exposure	0.447(0.183 to 1.092)	0.077	0.653 (0.233 to 1.834)	0.419
Usage of aluminum-containing antacids during pregnancy	3.694 (1.807 to 7.554)	0.0003**	2.338 (1.012 to 5.399)	0.047**
Multivitamin usage during pregnancy	0.386 (0.165 to 0.902)	0.028**	0.351 (0.127 to 0.972)	0.044**
Folic acid usage during pregnancy	0.3898 (0.168 to 0.907)	0.029**	0.474 (0.183 to 1.23)	0.125
Medication usage	0.711 (0.282 to 1.793)	0.470	0.632 (0.195 to 2.046)	0.444
Vaccination during pregnancy	0.22 (0.026 to 1.843)	0.163	0.185 (0.21 to 1.615)	0.127
Caffeine usage	1.689 (0.681 to 4.189)	0.258	1.833 (0.57 to 5.89)	0.310
Baking powder	0.437 (0.214 to 0.892)	0.0238**	0.235 (0.092 to 0.603)	0.003**
Microwave usage	0.958 (0.483 to 1.903)	0.903	0.689 (0.308 to 1.541)	0.364
Noise pollution (loud noise)	2.543 (1.113 to 5.811)	0.027**	2.659 (1.005 to 7.034)	0.049**
Owning pets	1.395 (0.556 to 3.503)	0.478	1.139 (0.357 to 3.633)	0.826

Neonatal factors 

Several neonatal factors during and after delivery have shown statistically significant results related to the development of autism. Among these neonatal factors, an increased risk of autism was seen in neonates whose birth weight was less than 2500g (aOR 4.19; 95% CI 1.16 - 14.84), male neonates (aOR 3.26; 95% CI 1.31 - 8.90), and neonates exposed to CT or MRI during the first year of life (aOR 6.94; 95% CI 1.15 - 42.07). Neonates taking iron supplements during the first six months of life demonstrated a decreased risk of autism (aOR 0.38; 95% 0.16 - 0.91). The following factors showed no statistical significance in autism development: delivery during winter months, difficulties during delivery, such as oxygen deprivation, neonatal jaundice, taking vitamin D and anti-reflux medications during the first six months of life, and having lactose intolerance or colic pain during the first six months of life (P ≥ 0.05). Table 3 displays the neonatal factors.

**Table 3:** Neonatal Factors **: P<0.05 is considered statistically significant. CI: Confidence Interval, CT: Computed Tomography, MRI: Magnetic Resonance Imaging

Risk Factor	Unadjusted Odds Ratio (95% CI)	P-Value	Adjusted Odds Ratio (95%CI)	P-Value
Cesarean delivery	0.476 (0.228 to 0.991)	0.047**	0.448 (0.184 to 1.093)	0.078
Birth weight (<2500g)	2.241 (0.732 to 6.858)	0.157	4.194 (1.185 to 14.841)	0.026**
Male gender	2.826 (1.31 to 6.098)	0.008**	3.259 (1.311 to 8.098)	0.011**
Born during winter months (December, January, February)	1.146 (0.538 to 2.439)	0.725	0.753 (0.294 to 1.925)	0.553
Neonatal jaundice	0.903 (0.434 to 1.878)	0.784	0.664 (0.276 to 1.597)	0.360
Difficulties at delivery (e.g. oxygen deprivation)	2.60 (0.869 to 7.782)	0.088	2.398 (0.663 to 8.669)	0.182
Incubated during the first month of life	2.513 (1.021 to 6.187)	0.045**	2.108 (0.735 to 6.044)	0.165
CT or MRI exposure during the first year of life	5.854 (1.503 to 22.803)	0.011**	6.942 (1.145 to 42.073)	0.035**
Taking vitamin D during the first six months of life	0.714 (0.356 to 1.43)	0.343	0.542 (0.242 to 1.214)	0.137
Taking iron during the first six months of life	0.492 (0.239 to 1.012)	0.054	0.384 (0.161 to 0.912)	0.030**
Taking anti-reflux medications during the first six months of life	0.64 (0.119 to 3.425)	0.602	0.75 (0.121 to 4.656)	0.757
Lactose intolerance diagnosed during the first six months of life	0.83 (0.073 to 9.39)	0.880	0.6 (0.043 to 8.326)	0.704
Colic pain during first six months of life	1.452 (0.645 to 3.269)	0.368	1.65 (0.654 to 4.167)	0.289

Parental factors 

Some parental factors were related to the development of autism in their offspring. An increased risk of autism was seen in mothers with ≥ two previous miscarriages (aOR 4.19; 95% CI 1.17 -14.97). Fathers’ ages and mothers giving birth in a country other than North Cyprus revealed no statistically significant results (P ≥ 0.05). Table 4 shows the parental factors.

**Table 4:** Parental Factors **: P<0.05 is considered statistically significant. CI: Confidence Interval

Risk Factor	Unadjusted Odds Ratio (95% CI)	P-Value	Adjusted Odds Ratio (95%CI)	P-Value
Father’s age at delivery *(≤ 30 years)*	1.931 (0.962 to 3.877)	0.064	1.515 (0.583 to 3.941)	0.394
Mother's age at delivery *(≤ 30 years)*	2.426 (1.156 to 5.088)	0.019**	2.637 (0.97 to 7.173)	0.058
Mother's country of birth is different than North Cyprus	1.141 (0.481 to 2.705)	0.765	0.729 (0.26 to 2.048)	0.549
Previous miscarriage (≥2)	3.862 (1.262 to 11.822)	0.018**	4.187 (1.172 to 14.967)	0.028**
Use of oral contraceptive pills (3 years before pregnancy)	3.682 (1.049 to 12.917)	0.042**	1.663 (0.403 to 6.873)	0.482

## Discussion

Our case-control study was able to establish various neonatal and maternal risk factors for the development of ASD. When considering maternal factors, compared to their control counterparts, significant risk factors for autism were observed in mothers with mental disorders, mothers with medical conditions, mothers using aluminum-containing antacids, and mothers exposed to loud noises while pregnant. On the other hand, protective factors were multivitamins usage during pregnancy, small amounts of baking powder usage during pregnancy, and mothers with threatened abortion. Neonatal factors implicated in the development of ASD were male gender, birth weight below 2500g, and exposure to MRI or CT in the first year of life.

Several meta-analyses and review articles were found in the literature combining multiple case-control studies related to ASD development. Studies that showed similar findings to our results include a meta-analysis done by Chen et al. (2016), which revealed that maternal autoimmune disease is a significant risk factor for ASD development with a pooled OR of 1.34 (95% CI 1.23 - 1.46) [12]. Maternal depression was also found to be a substantial risk factor for ASD in a meta-analysis performed by Kobayashi et al. (2016), especially in mothers using selective serotonin reuptake inhibitors (SSRIs) throughout their pregnancy with a pooled OR of 1.45 (95% CI 1.15 - 1.82) [13]. Moreover, Gardener et al. (2011) showed an increased risk of autism in neonates who have low birth weights (<2500g) with a pooled OR at 1.63 (95% CI 1.19 - 2.33), showing similarity to our findings [6]. Another meta-analysis was done by Wang et al. (2017) showed that male gender is associated with increased risk of autism with a relative risk (RR) of 1.47 (95% CI: 1.39 -1.55), a clear correlation that was also demonstrated in our study [7].

Wang et al. (2017) revealed that cigarette smoking was a non-significant factor [7]. However, Zhang et al. (2010) argued that several chemicals involved in second-hand smoking, such as polycyclic aromatic hydrocarbons and metals, have adverse health effects in fetal hypoxia and brain development [14]. These effects might hypothetically affect the social development of autistic children; nonetheless, our study did not show a relation to smoke exposure. Another systematic review was done by Cheng et al. (2019) showed that low birth weights in neonates together with autoimmune diseases and maternal depression increase the risk of developing ASD, further supporting our results [8]. Additionally, in the review of Elsabbagh (2019), there was some evidence of protective effects of multivitamins supplementation, including the use of folic acid and vitamin D, against the development of ASD [15]. Other studies also showed that iron, vitamin D, and fatty acids played a protective role against ASD development [16]. A meta-analysis by Sulaiman et al. (2020) demonstrated significant relation between all the metals and autism. Aluminum levels in blood were negatively associated with ASD, while aluminum levels in hair and urine were positively associated [17], focusing the lights on the effects of aluminum-containing anti-acids and their relation to autism found in our study. The majority of the studies mentioned above showed similarities with our study findings.

The meta-analysis done by Wu et al. (2016) focused solely on finding a connection between ASD and parental age. The findings differed from our study, as they showed a significant risk of developing ASD with increased parental ages [18]. A 10-year increase in paternal and maternal ages was associated with 21%, and 18% increased risk of autism in the offspring, with OR 1.21 (95% CI 1.18-1.24) and OR 1.18 (95% CI 1.10 -1.26), respectively [18]. In the meta-analysis of Curran et al. (2015), cesarean section was associated with increased risk of ASD with pooled aOR at 1.23 (95% CI 1.07 - 1.40) [19], compared to the insignificant results in our study. According to Xu et al. (2014), mothers with diabetes mellitus (DM) were shown to have an increased risk of having an autistic child compared to mothers without DM with a pooled RR of 1.48 (95% CI 1.25 to 1.75) [20]. Wang et al. (2017) also showed that gestational DM and gestational hypertension, in addition to cesarean delivery, were associated with increased ASD risk in the offspring, showing different findings from the results obtained in our study [7]. Additionally, Wang et al. demonstrated that threatened abortion actually increased the risk of autism with RR 1.54 (95% CI 1.28 - 1.87) [7], in contrast to our finding that showed it to be a protective factor. This equivocal finding in our study might be because women with threatened abortions in North Cyprus tend to be more cautious throughout their pregnancy and their delivery method. Some perinatal factors were also proven to be significantly related to ASD development, including hypoxic-ischemic damage, fetal distress during delivery, low Apgar (appearance, pulse, grimace, activity, and respiration) score, preterm birth, hyperbilirubinemia, small for gestational age, and congenital disabilities [6, 15, 21]. These findings were not demonstrated to be significant in our study.

Limitations

The differences in the results found in our study compared to literature might be attributed to the smaller sample size used, as opposed to the wider sample sizes used in the meta-analyses. Additionally, most of the meta-analyses included multiple pieces of research from different geographical areas, allowing the inclusion of various factors related to the studied population. A major limitation of our study is its retrospective design, predisposing it to recall bias. Prenatal, obstetric, and postnatal characteristics were evaluated based on surveys answered by mothers depending on their ability to remember past events. Furthermore, matching was not accomplished in our study due to the small sample size of the Northern Cyprus population, making it difficult to find many autistic children of similar ages. The ages of autistic children included in our study were increased to include children up to 18 years of age, aiming to reach larger numbers of autistic children. One difficulty that may also arise when exploring this topic is that many of these risk factors are connected, causing a possible confounder effect; mothers with medical conditions usually give birth to underweight neonates, pinpointing which risk factor is the most significant remains a challenge.

## Conclusions

Autism is one of the leading mental causes of disability in children worldwide. Looking at potential risk factors might give us insight into the possible preventative measures. Our study among children in Northern Cyprus showed evidence suggesting several maternal and neonatal factors associated with autism. The results showed increased odds of developing ASD in mothers with mental disorders such as depression and anxiety, mothers with medical conditions such as Hashimoto’s thyroiditis, mothers using aluminum-containing anti-acids, mothers exposed to loud noises during pregnancy, mothers with ≥ two previous miscarriages, neonates with birth weight <2500 grams, male gender neonates, and neonates exposed to MRI or CT during their first year of life. The factors that demonstrated decreased odds of ASD development were observed in mothers using multivitamins during pregnancy, mothers consuming slight amounts of baking powder during pregnancy, mothers with threatened abortion, and neonates taking iron supplementation during the first six months of life. Although there is some evidence to suggest that exposure to specific factors during the prenatal or postnatal periods may increase or decrease ASD risk, there is insufficient evidence that implicates a specific aetiological factor for autism. It is recommended that future studies be performed on a larger scale to support further the factors associated with ASD development.

## Appendices

**Table 5:** STROBE statement—Checklist of items that should be included in reports of case-control studies STROBE - STrengthening the Reporting of OBservational studies in Epidemiology

	Item Number	Recommendations	The corresponding text from our manuscript
Title and Abstract	1	(a) Indicate the study’s design with a commonly used term in the title or the abstract	“Evaluation of the Relationship between Environmental Factors, Nutrition, and Metabolic Changes in Children Diagnosed with Autism in North Cyprus: A Case-Control Study”
		(b) Provide in the abstract an informative and balanced summary of what was done and what was found	“We conducted an unmatched case-control study composed of 56 autistic cases and 85 control children in North Cyprus. Cases recruited were previously diagnosed by a pediatric psychiatrist as being on the autistic spectrum. Parental questionnaires were distributed, and the collected data were analyzed using IBM SPSS version 24. Binary logistic regression was used to compute the adjusted odds ratios (aOR), adjusted for possible confounders.” “Our results showed increased odds of developing ASD in mothers with mental disorders such as depression and anxiety *(aOR 6.99; 95% CI 1.94 - 25.24)*, mothers with medical conditions such as Hashimoto’s thyroiditis *(aOR 2.68; 95% CI 1.06 - 6.78)*, mothers using aluminum-containing anti-acids *(aOR 2.34; 95% CI 1.012 - 5.39)*, mothers exposed to loud noises during pregnancy *(aOR 2.66; 95% CI 1.005 - 7.034)*, mothers with ≥ two previous miscarriages (aOR 4.19; 95% CI 1.17 -14.97), neonates with birth weight <2500 grams (aOR 4.19; 95% CI 1.16 - 14.84), male gender neonates (aOR 3.26; 95% CI 1.31 - 8.90), and neonates exposed to MRI or CT during their first year of life (aOR 6.94; 95% CI 1.15 - 42.07). The factors that demonstrated decreased odds of ASD development were observed in mothers using multivitamins during pregnancy *(aOR 0.35; 95% CI 0.13 - 0.97)*, mothers consuming slight amounts of baking powder during pregnancy *(aOR 0.235; 95% CI 0.09 - 0.60)*, mothers with threatened abortion *(aOR 0.35; 95% CI 0.12 - 0.98)*, and neonates taking iron supplementation during the first six months of life (aOR 0.38; 95% 0.16 - 0.91).”
Introduction			
background/ rationale	2	Explain the scientific background and rationale for the investigation being reported	“Despite being a common condition with a lifelong course, ASD showed no apparent connection with a single specific risk factor [2]. Instead, a combination of both genetic and environmental factors is suggested [5].” “Identifying the possible risk factors linked to ASD is essential in preventing the condition. In addition to the substantial economic burden of ASD, the quality of life for affected individuals and their families is decreased substantially. Therefore, targeting disease prevention is a crucial element in improving the quality of life in recent years.”
Objectives	3	State specific objectives, including any prespecified hypotheses	“Our study investigated the relationship between autism and prenatal, perinatal, and postnatal conditions to suggest possible risk factors and protective factors for ASD”
Methods			
Study design	4	Present key elements of study design early in the paper	“We conducted a case-control study in the Turkish Republic of Northern Cyprus in 2017 among children under the age of 18.”
Setting	5	Describe the setting, locations, and relevant dates, including periods of recruitment, exposure, follow-up, and data collection	“We conducted a case-control study in the Turkish Republic of Northern Cyprus in 2017 among children under the age of 18.” “We obtained ethical approval from the “Ethics Committee of Eastern Mediterranean University”. In addition, informed consent was obtained from the parents of autistic children by visiting eight special education centers in North Cyprus and calling the parents of autistic children registered in those centers. We then distributed hard-copy questionnaires to be filled by the mothers of autistic children and collected the questionnaires one month later. We also obtained informed consent from the mothers of controlled children by reaching out to two public schools in North Cyprus and distributing the questionnaires to the heads of those public schools. The questionnaire was distributed in the Turkish language and was developed based on a thorough literature review of similar studies”.
Participants	6	(a) Give the eligibility criteria, and the sources and methods of case ascertainment and control selection. Give the rationale for the choice of cases and controls	“We estimated the required sample size of cases as 56 cases using the "open epi" website for unmatched case-control studies [10]. The term "cases" refers to children who were diagnosed as being autistic by a pediatric psychiatrist in North Cyprus, based on the Diagnostic and Statistical Manual of Mental Disorders, 5th Edition (DSM-5) criteria [11]. The term “controls” refers to children without a diagnosis of autism who were enrolled in public schools.”
		(b) For matched studies, give matching criteria and the number of controls per case	This study was an unmatched case-control study.
Variables	7	Clearly define all outcomes, exposures, predictors, potential confounders, and effect modifiers. Give diagnostic criteria, if applicable	“The demographic variables in our study include gender, child’s age, mother’s and father’s ages at the birth of their child, child’s order, birth weight, birth month, mother’s country of birth, living in an old house, mother having medical conditions, and previous miscarriages. The other part of the survey focused on the possible risk factors associated with the development of autism during the prenatal, perinatal, and postnatal periods”. “The odds ratio was adjusted for potential confounders, including mother’s age, father’s age, child’s gender, birth weight, previous miscarriages, mother’s medical conditions, and pregnancy-induced medical conditions. These confounders were chosen based on data from previous literature studies.”
Data sources/ measurement	8*	For each variable of interest, give sources of data and details of methods of assessment (measurement). Describe comparability of assessment methods if there is more than one group	“We then distributed hard-copy questionnaires to be filled by the mothers of autistic children” “The questionnaire was distributed in the Turkish language and was developed based on a thorough literature review of similar studies. The questionnaire asked yes-no questions about the possible exposure to each variable during the pregnancy period.”
Bias	9	Describe any efforts to address potential sources of bias	“The odds ratio was adjusted for potential confounders, including mother’s age, father’s age, child’s gender, birth weight, previous miscarriages, mother’s medical conditions, and pregnancy-induced medical conditions. These confounders were chosen based on data from previous literature studies.”
Study size	10	Explain how the study size was arrived at	“According to the Statistical Yearbook of Northern Cyprus published in February 2016, the total number of children in North Cyprus under 18 years old was 73,500, with an average of 4000 new births per year [9]. Approximately 300 children were enrolled in special education centers in North Cyprus, and around 100 of them were diagnosed as having an autism spectrum disorder. Based on the given numbers, we estimated the required sample size of cases as 56 cases using the "open epi" website for unmatched case-control studies [10].”
Quantitative variables	11	Explain how quantitative variables were handled in the analyses. If applicable, describe which groupings were chosen and why	“We compared the distribution of demographics and general characteristics between autistic and non-autistic children by performing Pearson’s chi-square test, as well as independent sample t-test for quantitative variables including child’s age, mother’s age, father’s age, and order of the child.”
Statistical methods	12	(a) Describe all statistical methods, including those used to control for confounding	“Data analysis was done through the IBM Statistical Package for the Social Sciences (SPSS) version 24 software, and statistical significance was set at a two-sided P-value of <0.05. Descriptive statistics were computed on all variables as the primary method of data evaluation. We compared the distribution of demographics and general characteristics between autistic and non-autistic children by performing Pearson’s chi-square test, as well as independent sample t-test for quantitative variables including child’s age, mother’s age, father’s age, and order of child. We also used the binary logistic regression analysis with a 95% Confidence Interval (CI) to calculate the unadjusted odds ratio (OR) and the adjusted odds ratios (aOR) for potential confounders.”
		(b) Describe any methods used to examine subgroups and interactions	No subgroups were examined
		(c) Explain how missing data were addressed	“The statistical analysis on SPSS was done using the number of responses received for each variable, and the missing responses were not included in our statistical calculations.”
		(d) If applicable, explain how matching of cases and controls was addressed	No matching was done
		(e) Describe any sensitivity analyses	No sensitivity analysis was done
Results			
Participants	13*	(a) Report numbers of individuals at each stage of study—eg numbers potentially eligible, examined for eligibility, confirmed eligible, included in the study, completing follow-up, and analysed	“The total sample size included in our study comprised 141 patients - 56 cases and 85 controls. The potentially eligible number of autistic cases in North Cyprus was estimated to be 100 cases; out of these cases, we managed to collect responses from the mothers of 56 autistic children, which was the number of cases suggested by “openepi website”. We also distributed 100 questionnaires for the control group, and we managed to collect responses from the mothers of 85 controlled children.
		(b) Give reasons for non-participation at each stage	Reasons for non-participation in this study included parental preferences, as some parents did not want to feel guilty for potentially causing disease to their children.
		(c) Consider use of a flow diagram	No flow diagram was used
Descriptive data	14*	(a) Give characteristics of study participants (eg demographic, clinical, social) and information on exposures and potential confounders	Table 1: Demographical Characteristics
		(b) Indicate number of participants with missing data for each variable of interest	Table 1: Demographical Characteristics
Outcome data	15*	Report numbers in each exposure category, or summary measures of exposure	Table 2: Maternal Factors During Pregnancy, Table 3: Neonatal Factors, Table 4: Parental Factors
Main results	16	(a) Give unadjusted estimates and, if applicable, confounder-adjusted estimates and their precision (eg, 95% confidence interval). Make clear which confounders were adjusted for and why they were included	Table 2: Maternal Factors During Pregnancy, Table 3: Neonatal Factors, Table 4: Parental Factors, “Potential confounders were chosen based on data from previous literature studies. Adjusted confounders in our study included mother’s age, father’s age, child’s gender, birth weight, previous miscarriages, mother’s medical conditions, and pregnancy-induced medical conditions.”
		(b) Report category boundaries when continuous variables were categorized	“Continuous variables that were categorized included birth weight, which was categorized into <2500g and >2500g; mother’s and father’s ages at delivery, which were categorized into <30 years and >30 years of age; and the birth month, which was categorized into winter months(December, January, February) and non-winter months.”
		(c) If relevant, consider translating estimates of relative risk into absolute risk for a meaningful time period	Absolute risk was not calculated.

**Table 6:** STROBE statement continued—Checklist of items that should be included in reports of case-control studies STROBE - STrengthening the Reporting of OBservational studies in Epidemiology

	Item No	Recommendation	The corresponding text from our manuscript
Other analyses	17	Report other analyses done—eg analyses of subgroups and interactions, and sensitivity analyses	Descriptive statistics, Chi square test, independent sample t-test, and binary logistic regression for odds ratio.
Discussion			
Key results	18	Summarise key results with reference to study objectives	“This case-control study was able to establish various neonatal and maternal risk factors for the development of ASD. When considering maternal factors, compared to their control counterparts, significant risk factors for autism were observed in mothers with mental disorders, mothers with medical conditions, mothers using aluminum-containing antacids, and mothers exposed to loud noises while pregnant. On the other hand, protective factors were multivitamins usage during pregnancy, small amounts of baking powder usage during pregancy, and mothers with threatened abortion. Neonatal factors implicated in the development of ASD were male gender, birth weight below 2500g and exposure to MRI or CT in the first year of life.”
Limitations	19	Discuss limitations of the study, taking into account sources of potential bias or imprecision. Discuss both direction and magnitude of any potential bias	“A major limitation of our study is its retrospective design, predisposing it to recall bias. Prenatal, obstetric, and postnatal characteristics were evaluated based on surveys answered by mothers depending on their ability to remember past events. Furthermore, matching was not accomplished in our study due to the small sample size of the Northern Cyprus population, making it difficult to find many autistic children of similar ages.”
Interpretation	20	Give a cautious overall interpretation of results considering objectives, limitations, multiplicity of analyses, results from similar studies, and other relevant evidence	“...The majority of the studies mentioned above showed similarities with our study findings.” The meta-analysis done by Wu et al. (2016) focused solely on finding a connection between ASD and parental age. The findings differed from our study, as they showed a significant risk of developing ASD with increased parental ages [18]. A 10-year increase in paternal and maternal ages was associated with 21% and 18% increased risk of autism in the offspring, with OR 1.21(95% CI 1.18-1.24) and OR 1.18 (95% CI 1.10 -1.26), respectively [18]. In the meta-analysis of Curran et al. (2015), cesarean section was associated with increased risk of ASD with pooled aOR at 1.23 (95% CI 1.07 - 1.40) [19], compared to the insignificant results in our study. According to Xu et al. (2014), mothers with diabetes mellitus (DM) were shown to have an increased risk of having an autistic child compared to mothers without DM with a pooled RR of 1.48 (95% CI 1.25 to 1.75) [20]. Wang et al. (2017) also showed that gestation DM and gestational hypertension in addition to cesarean delivery were associated with increased ASD risk in the offspring, showing different findings from the results obtained in our study [7]. Additionally, Wang et al. demonstrated that threatened abortion actually increased the risk of autism with RR 1.54 (95% CI 1.28 - 1.87) [7], in contrast to our finding that showed it to be a protective factor. This equivocal finding in our study might be due to the fact that women with threatened abortion in North Cyprus tend to be more cautious throughout their pregnancy and their method of delivery. Some perinatal factors were also proven to be significantly related to ASD development, including hypoxic-ischemic damage, fetal distress during delivery, low Apgar score, preterm birth, hyperbilirubinemia, small for gestational age, and birth defects [6, 15, 21]. These findings were not demonstrated to be significant in our study.
Generalisability	21	Discuss the generalisability (external validity) of the study results	“Several meta-analyses and review articles were found in the literature combining multiple case-control studies related to ASD development. Studies that showed similar findings to our results include a meta-analysis done by Chen et al. (2016), which revealed that maternal autoimmune disease is a significant risk factor for ASD development with a pooled OR of 1.34 (95% CI 1.23 - 1.46) [12]. Maternal depression was also found to be a substantial risk factor for ASD in a meta-analysis performed by Kobayashi et al. (2016), especially in mothers using selective serotonin reuptake inhibitors (SSRIs) throughout their pregnancy with a pooled OR of 1.45 (95% CI 1.15 - 1.82) [13]. Moreover, Gardener et al. (2011) showed an increased risk of autism in neonates who have low birth weights (<2500g) with a pooled OR at 1.63 (95% CI 1.19 - 2.33), showing similarity to our findings [6]. Another meta-analysis was done by Wang et al. (2017) showed that male gender is associated with increased risk of autism with a relative risk (RR) of 1.47 (95% CI: 1.39 -1.55), a clear correlation that was also demonstrated in our study [7]. Wang et al. (2017) revealed that cigarette smoking was a non-significant factor [7].” “Another systematic review was done by Cheng et al. (2019) showed that low birth weights in neonates together with autoimmune diseases and maternal depression increase the risk of developing ASD, further supporting our results [8]. Additionally, in the review of Elsabbagh (2019), there was some evidence of a protective effect of multivitamins supplementation, including the use of folic acid and vitamin D, against the development of ASD [15]. Other studies also showed that iron, vitamin D, and fatty acids played a protective role against ASD development [16].”
Other information			
Funding	22	Give the source of funding and the role of the funders for the present study and, if applicable, for the original study on which the present article is based	No funding source was allocated.

## References

[1] (2021). Autism spectrum disorders. https://www.who.int/news-room/fact-sheets/detail/autism-spectrum-disorders.

[2] (2021). Autism statistics and facts. https://www.autismspeaks.org/autism-statistics-asd.

[3] (2021). What is autism?. https://www.autismspeaks.org/what-autism.

[4] (2021). Autism spectrum disorder (ASD). https://www.cdc.gov/ncbddd/autism/facts.html.

[5] (2021). Autism spectrum disorder. https://www.mayoclinic.org/diseases-conditions/autism-spectrum-disorder/symptoms-causes/syc-20352928.

[6] Gardener H, Spiegelman D, Buka SL (2011). Perinatal and neonatal risk factors for autism: a comprehensive meta-analysis. Pediatrics.

[7] Wang C, Geng H, Liu W, Zhang G (2017). Prenatal, perinatal, and postnatal factors associated with autism: a meta-analysis. Medicine (Baltimore).

[8] Cheng J, Eskenazi B, Widjaja F, Cordero JF, Hendren RL (2019). Improving autism perinatal risk factors: a systematic review. Med Hypotheses.

[9] (2017). TRNC state planning organization. http://www.devplan.org/index.html.

[10] (2017). Open source statistics for public health. http://wwww.openepi.com/SampleSize/SSCC.htm.

[11] (2021). Autism diagnosis criteria: DSM-5. https://www.autismspeaks.org/autism-diagnosis-criteria-dsm-5.

[12] Chen SW, Zhong XS, Jiang LN (2016). Maternal autoimmune diseases and the risk of autism spectrum disorders in offspring: a systematic review and meta-analysis. Behav Brain Res.

[13] Kobayashi T, Matsuyama T, Takeuchi M, Ito S (2016). Autism spectrum disorder and prenatal exposure to selective serotonin reuptake inhibitors: a systematic review and meta-analysis. Reprod Toxicol.

[14] Zhang X, Lv CC, Tian J, Miao RJ, Xi W, Hertz-Picciotto I, Qi L (2010). Prenatal and perinatal risk factors for autism in China. J Autism Dev Disord.

[15] Elsabbagh M (2020). Linking risk factors and outcomes in autism spectrum disorder: is there evidence for resilience?. BMJ.

[16] Peretti S, Mariano M, Mazzocchetti C, Mazza M, Pino MC, Verrotti Di Pianella A, Valenti M (2019). Diet: the keystone of autism spectrum disorder?. Nutr Neurosci.

[17] Sulaiman R, Wang M, Ren X (2020). Exposure to aluminum, cadmium, and mercury and autism spectrum disorder in children: a systematic review and meta-analysis. Chem Res Toxicol.

[18] Wu S, Wu F, Ding Y, Hou J, Bi J, Zhang Z (2017). Advanced parental age and autism risk in children: a systematic review and meta-analysis. Acta Psychiatr Scand.

[19] Curran EA, O'Neill SM, Cryan JF, Kenny LC, Dinan TG, Khashan AS, Kearney PM (2015). Research review: birth by caesarean section and development of autism spectrum disorder and attention-deficit/hyperactivity disorder: a systematic review and meta-analysis. J Child Psychol Psychiatry.

[20] Xu G, Jing J, Bowers K, Liu B, Bao W (2014). Maternal diabetes and the risk of autism spectrum disorders in the offspring: a systematic review and meta-analysis. J Autism Dev Disord.

[21] Guinchat V, Thorsen P, Laurent C, Cans C, Bodeau N, Cohen D (2012). Pre-, peri- and neonatal risk factors for autism. Acta Obstet Gynecol Scand.

